# Concerted down-regulation of immune-system related genes predicts metastasis in colorectal carcinoma

**DOI:** 10.1186/1471-2407-14-64

**Published:** 2014-02-05

**Authors:** Marion Fehlker, Matthew R Huska, Thomas Jöns, Miguel A Andrade-Navarro, Wolfgang Kemmner

**Affiliations:** 1 Translational Oncology, Experimental Clinical Research Center, Charite Campus Buch, Lindenberger Weg 80, D-13125 Berlin, Germany; 2 Computational Biology and Data Mining group, Max Delbrueck Center for Molecular Medicine, Robert Roessle Str. 10, D-13125 Berlin, Germany; 3 Presently at Department for Computational Molecular Biology, Max Planck Institute for Molecular Genetics, 14195 Berlin, Germany; 4 Institut für Integrative Anatomie, Charite Campus Mitte, Philippstr. 12, D-10115 Berlin, Germany

**Keywords:** Early colorectal cancer, Metastasis, Computational marker analysis, Immune system, Gene expression profiling

## Abstract

**Background:**

This study aimed at the identification of prognostic gene expression markers in early primary colorectal carcinomas without metastasis at the time point of surgery by analyzing genome-wide gene expression profiles using oligonucleotide microarrays.

**Methods:**

Cryo-conserved tumor specimens from 45 patients with early colorectal cancers were examined, with the majority of them being UICC stage II or earlier and with a follow-up time of 41–115 months. Gene expression profiling was performed using Whole Human Genome 4x44K Oligonucleotide Microarrays. Validation of microarray data was performed on five of the genes in a smaller cohort.

**Results:**

Using a novel algorithm based on the recursive application of support vector machines (SVMs), we selected a signature of 44 probes that discriminated between patients developing later metastasis and patients with a good prognosis. Interestingly, almost half of the genes was related to the patients’ immune response and showed reduced expression in the metastatic cases.

**Conclusions:**

Whereas up to now gene signatures containing genes with various biological functions have been described for prediction of metastasis in CRC, in this study metastasis could be well predicted by a set of gene expression markers consisting exclusively of genes related to the MHC class II complex involved in immune response. Thus, our data emphasize that the proper function of a comprehensive network of immune response genes is of vital importance for the survival of colorectal cancer patients.

## Background

Colorectal cancer (CRC) is one of the most common cancers with an annual incidence of more than 400,000 new cases and 212,000 deaths in Europe alone [1]. Treatment decisions are based on histopathological staging of the primary tumor, considering depth of tumor infiltration and metastatic spread to lymph nodes or distant organs. Therapy consists of surgical resection of the tumor and additional chemo- or/and radiotherapy in the case of lymph node or distant metastases. Approximately 40% of patients with CRC die within 5 years due to local recurrence or distant metastases. However, histopathological staging of the primary tumor does not allow for outcome prediction, resulting in under- as well as over-treatment of particular groups of patients.

The heterogeneity of patients with complex diseases where the phenotype can have multiple genetic and environmental components [2,3] usually prevents one from finding differentially expressed genes between different groups of patients. Therefore, recent studies aimed at outcome prediction based on gene expression [4-10], often resulting in a diverse set of genes without a common biological function. In such a situation, specialized methods of analysis for differential gene expression are required. In particular, Zhang *et al*. [11] developed a method appropriate for clinical data that iteratively selects groups of genes that are optimized for discriminating between different groups of patients, with patient numbers similar to those in this study. Unlike methods that attempt to select individual genes by their significant differential expression between classes of samples (e.g., SAM [12]), the gene set is selected as a set of markers that discriminate when used collectively but not necessarily when each gene is used individually. Using this methodology we were able to identify a predictive gene signature in an unbiased manner that shows a functional relationship to tumor biology. Surprisingly, most of the genes that were found to be informative for patients’ metastasis were related to the immune system but not to common tumor cell characteristics such as angiogenesis, adhesion or invasion.

## Methods

### Patients

This study comprises only patients with early CRC of UICC stages I-III, with the majority of them (76%) being UICC stage II or earlier (Table 1). Inclusion criteria were: CRC, R0 resection and follow-up of at least three years or until disease recurrence. Exclusion criteria were: presence of a secondary carcinoma, patient age less than 40 years at the time of surgery, local recurrence, preoperative treatment and radiotherapy.

**Table 1:** Patient characteristics

**Characteristic**	**Non-recurrent**		**Metachronous metastasis**		**p-value**^**1**^
	**absolute**	***%***	**absolute**	***%***	
**Total number**	33		12		
**Age [years]**					n.s.
Mean	65		68		
Median	67		67		
**Disease-free survival time [months]**					0.000
Mean	78		17		
Median	77		13		
**Gender**					n.s.
Female	14	*42,4*	8	*66,7*	
Male	19	*57,6*	4	*33,3*	
**UICC stage**					n.s.
I	6	*18,2*	2	*16,7*	
II	18	*54,5*	8	*66,7*	
III	9	*27,3*	2	*16,7*	
**Location**					n.s.
Colon	14	*42,4*	5	*41,7*	
Sigma	16	*48,5*	4	*33,3*	
Rectum	3	*9,1*	3	*25*	

^1^The given p-value refers to the significance of the correlation of the respective characteristic with disease-free survival time and was calculated using a two-sided t-test. n.s., not significant.

### Specimen characteristics

Cryo-conserved tumor specimens from 45 patients and follow-up data were obtained from the Tumorbank of the Charité Comprehensive Cancer Center (CCCC), Charité Campus Buch, Berlin. Tissue samples from patients that had undergone resection for sporadic colorectal adenocarcinoma at Charité Robert Roessle Hospital (1995–2006) were enrolled for this study (after informed consent). The study was approved by the local ethics committee (Charité Universitätsmedizin Berlin). Archived primary tumor tissues were collected immediately after surgical removal and snap-frozen in liquid nitrogen according to internal protocols. In addition to routine pathological examination of the tumor tissue, histopathology of each sample used for experimental analysis was reviewed by an experienced pathologist to confirm diagnosis, tissue composition and tumor content. Only non-necrotic tissue samples lacking fatty or connective tissue and composed of at least 60% tumor cells (mean: 73%) were processed. Disease-free time was defined as the time period from the date of surgery to confirmed distant tumor metastasis date for metastatic cases and from the date of surgery to the date of last follow-up for non-recurrent patients.

### Gene expression analysis

Total RNA was extracted from each frozen tumor specimen using the RNeasy Mini Prep Kit (Qiagen, Hilden, Germany). RNA quality was checked by Bioanalyzer (Agilent, Santa Clara, CA, USA). Only RNA samples showing an RNA Integrity Number (RIN) of at least 7.0 were used for labelling. Total RNA (1 μg) was labeled with Cy3 using the Low Input RNA Amplification Kit (Agilent, Santa Clara, CA, USA). Labeled cRNAs were hybridized to Whole Human Genome 4x44K Oligonucleotide Microarrays (Agilent, Santa Clara, CA, USA) following the manufacturer´s protocols. Arrays were scanned by using standard Agilent protocols and a G2565AA Microarray Scanner (Agilent, Santa Clara, CA, USA). Raw expression values were determined using Feature Extraction 8.0 software (Agilent, Santa Clara, CA, USA). Data were uploaded to GEODataSets with accession: GSE31905 and ID: 200031905 under the title: Gene expression profiling of colorectal carcinomas.

### Data analysis

Microarray data analysis was performed using R/Bioconductor [13]. Raw expression values were normalized using variance stabilization normalization [14], and array quality was evaluated with the arrayQualityMetrics library [15]. Univariate differential expression analysis was performed using methods from the limma library [13], and multiple testing was controlled using the Benjamini and Hochberg method [16]. Individual probes were considered differentially expressed if they had a fold change greater than 2.0 and a false discovery rate (q-value) less than 0.05. No significant differentially expressed genes between recurrent and non-recurrent samples were observed. Principal component analysis of the data indicated that the full vectors of gene expression could not be used to discriminate between sample classes (Additional file 1: Figure S1).

Multivariate feature selection was performed using the R-SVM algorithm [11]. The algorithm was configured to perform 100 bootstrapping cross-validation steps and the best 42 probes were saved from each step. Probes that were selected at least once were kept, yielding 44 probes that were considered for further analysis. We then filtered out any probes that did not have an associated gene symbol (Table 2). The resulting 30 probes were analyzed using the Bioconductor package GOstats [17], which identified the GO term “immune system process” (GO:0002376) associated to 14 of the genes as being significantly (p-value 4.6E-12) enriched (Table 3). ROC curves, Kaplan-Meier analysis and clinical measure statistics (Table 1) were calculated using SPSS 14 (SPSS, Chicago, IL, USA).

**Table 2:** Annotations of genes with prognostic value in the prediction of CRC metastasis

**Agilent probe name**	**Gene symbol**	**Gene name**	**RefSeq**	**GO immune**^**1**^
A_23_P138635	BNIP3	BCL2/adenovirus E1B 19 kDa interacting protein 3	NM_004052, NP_004043	Yes
A_23_P55270	CCL18	chemokine (C-C motif) ligand 18 (pulmonary and activation-regulated)	NM_002988, NP_002979	Yes
A_23_P70095	CD74	CD74 molecule, major histocompatibility complex, class II invariant chain	NM_001025158, NM_001025159, NM_004355, NP_001020329, NP_001020330, NP_004346	Yes
A_24_P131589	CD86	CD86 molecule	NM_006889, NM_175862, NP_008820, NP_787058	Yes
A_24_P510357	CKAP2	cytoskeleton associated protein 2	NM_001098525, NM_018204, NP_001091995, NP_060674	
A_23_P125278	CXCL11	chemokine (C-X-C motif) ligand 11	NM_005409, NP_005400	Yes
A_23_P18452	CXCL9	chemokine (C-X-C motif) ligand 9	NM_002416, NP_002407	Yes
A_23_P254944	GSTT1	glutathione S-transferase theta 1	NM_000853, NP_000844	
A_23_P42306	HLA-DMA	major histocompatibility complex, class II, DM alpha	NM_006120, NP_006111	Yes
A_23_P258769	HLA-DPB1	major histocompatibility complex, class II, DP beta 1	NM_002121, NP_002112	Yes
A_24_P370472	HLA-DRB4	major histocompatibility complex, class II, DR beta 4	NM_021983, NP_068818, XM_001723414, XM_001723417, XM_001723419, XP_001723466, XP_001723469, XP_001723471	Yes
A_23_P31006	HLA-DRB5	major histocompatibility complex, class II, DR beta 5	NM_002125, NP_002116	Yes
A_23_P112026	IDO1	indoleamine 2,3-dioxygenase 1	NM_002164, NP_002155	Yes
A_23_P119943	IGFBP2	insulin-like growth factor binding protein 2, 36 kDa	NM_000597, NP_000588	
A_23_P158817	IGHG1	immunoglobulin heavy locus		
A_24_P92683	IGHA1	immunoglobulin heavy constant alpha 1		Yes
A_24_P204727	IGHG1	immunoglobulin heavy constant gamma 1 (G1m marker)		
A_24_P315941	IGHG1	immunoglobulin heavy constant gamma 1 (G1m marker)		
A_23_P21249	IGHG1	immunoglobulin heavy constant gamma 1 (G1m marker)		
A_24_P519504	IGL@	immunoglobulin lambda locus		
A_24_P83102	IGLL1	immunoglobulin lambda-like polypeptide 1	NM_020070, NM_152855, NP_064455, NP_690594	Yes
A_23_P76249	KRT6B	keratin 6B	NM_005555, NP_005546	
A_23_P1691	MMP1	matrix metallopeptidase 1 (interstitial collagenase)	NM_002421, NP_002412	
A_23_P169494	ORM1	orosomucoid 1	NM_000607, NP_000598	Yes
A_23_P213508	PCSK1	proprotein convertase subtilisin/kexin type 1	NM_000439, NP_000430	
A_24_P174793	PCSK1	proprotein convertase subtilisin/kexin type 1	NM_000439, NP_000430	
A_23_P149517	PIGR	polymeric immunoglobulin receptor	NM_002644, NP_002635	
A_24_P844984	PIGR	polymeric immunoglobulin receptor	NM_002644, NP_002635	
A_23_P1962	RARRES3	retinoic acid receptor responder (tazarotene induced) 3	NM_004585, NP_004576	
A_23_P81898	UBD	ubiquitin D	NM_006398, NP_006389	

^1^genes affiliated to the GO term “immune system process”.

**Table 3:** GO-analysis of 44 signature genes

**Biological process GO ID**	**P-value**	**Odds ratio**	**Ex-pected**	**Count**	**Size**	**GO Term**	**Associated gene symbols**
GO:0006955	1.4E-12	31.69	1.04	13	620	Immune response	HLA-DMA, HLA-DPB1, HLA-DRB4, HLA-DRB5, IGHA1, IGLL1, IDO1, CXCL9, CCL18, CXCL11, BNIP3, CD86, CD74
GO:0002376	4.6E-12	26.94	1.47	14	874	Immune system process	HLA-DMA, HLA-DPB1, HLA-DRB4, HLA-DRB5, IGHA1, IGLL1, IDO1, CXCL9, ORM1, CCL18, CXCL11, BNIP3, CD86, CD74
GO:0002504	1.8E-08	208.89	0.03	4	18	Antigen processing and presentation of peptide or polysaccharide antigen via MHC class II	HLA-DMA, HLA-DPB1, HLA-DRB4, HLA-DRB5
GO:0019882	3.5E-08	73.05	0.10	5	58	Antigen processing and presentation	HLA-DMA, HLA-DPB1, HLA-DRB4, HLA-DRB5, CD74
GO:0050896	3.6E-06	8.20	4.13	14	2454	Response to stimulus	HLA-DMA, HLA-DPB1, HLA-DRB4, HLA-DRB5, IGHA1, IGLL1, IDO1, CXCL9, ORM1, CCL18, CXCL11, BNIP3, CD86, CD74
GO:0002828	4.0E-05	327.34	0.01	2	6	Regulation of T-helper 2 type immune response	IDO1, CD86
GO:0002682	6.7E-05	14.53	0.45	5	267	Regulation of immune system process	HLA-DMA, IDO1, ORM1, CD86, CD74
GO:0042092	1.2E-04	163.62	0.02	2	10	T-helper 2 type immune response	IDO1, CD86
GO:0006954	1.4E-04	12.44	0.52	5	310	Inflammatory response	IDO1, CXCL9, ORM1, CCL18, CXCL11
GO:0030217	1.5E-04	34.98	0.10	3	62	T cell differentiation	HLA-DMA, CD86, CD74
GO:0045058	1.8E-04	130.87	0.02	2	12	T cell selection	HLA-DMA, CD74
GO:0006952	2.2E-04	8.75	0.93	6	550	Defense response	IDO1, CXCL9, ORM1, CCL18, CXCL11, BNIP3
GO:0045582	3.2E-04	93.45	0.03	2	16	Positive regulation of T cell differentiation	HLA-DMA, CD86
GO:0045621	4.1E-04	81.76	0.03	2	18	Positive regulation of lymphocyte differentiation	HLA-DMA, CD86
GO:0002460	4.7E-04	23.13	0.16	3	92	Adaptive immune response based on somatic recombination of immune receptors built from immunoglobulin superfamily domains	HLA-DMA, IDO1, CD86
GO:0030098	4.7E-04	23.13	0.16	3	92	Lymphocyte differentiation	HLA-DMA, CD86, CD74
GO:0002250	4.9E-04	22.88	0.16	3	93	Adaptive immune response	HLA-DMA, IDO1, CD86
GO:0044419	5.8E-04	12.38	0.40	4	236	Interspecies interaction between organisms	HLA-DRB4, MMP1, BNIP3, CD86
GO:0009611	7.2E-04	8.52	0.75	5	445	Response to wounding	IDO1, CXCL9, ORM1, CCL18, CXCL11
GO:0002694	8.8E-04	18.52	0.19	3	114	Regulation of leukocyte activation	HLA-DMA, CD86, CD74
GO:0050865	1.0E-03	17.56	0.20	3	120	Regulation of cell activation	HLA-DMA, CD86, CD74
GO:0045580	1.1E-03	46.67	0.05	2	30	Regulation of T cell differentiation	HLA-DMA, CD86
GO:0051704	1.2E-03	7.57	0.84	5	498	Multi-organism process	HLA-DRB4, IDO1, MMP1, BNIP3, CD86
GO:0042110	1.4E-03	15.54	0.23	3	135	T cell activation	HLA-DMA, CD86, CD74
GO:0050776	1.7E-03	14.54	0.24	3	144	Regulation of immune response	HLA-DMA, IDO1, CD86
GO:0002822	1.8E-03	36.28	0.06	2	38	Regulation of adaptive immune response based on somatic recombination of immune receptors built from immunoglobulin superfamily domains	IDO1, CD86
GO:0045619	1.8E-03	36.28	0.06	2	38	Regulation of lymphocyte differentiation	HLA-DMA, CD86
GO:0002819	1.9E-03	35.29	0.07	2	39	Regulation of adaptive immune response	IDO1, CD86
GO:0002521	1.9E-03	13.94	0.25	3	150	Leukocyte differentiation	HLA-DMA, CD86, CD74
GO:0002684	1.9E-03	13.94	0.25	3	150	Positive regulation of immune system process	HLA-DMA, IDO1, CD86
GO:0006935	2.1E-03	13.57	0.26	3	154	Chemotaxis	CXCL9, CCL18, CXCL11
GO:0042330	2.1E-03	13.57	0.26	3	154	Taxis	CXCL9, CCL18, CXCL11
GO:0007267	2.3E-03	6.50	0.97	5	576	Cell-cell signaling	CXCL9, PCSK1, CCL18, CXCL11, CD86
GO:0050870	3.5E-03	25.58	0.09	2	53	Positive regulation of T cell activation	HLA-DMA, CD86
GO:0046649	4.2E-03	10.52	0.33	3	197	Lymphocyte activation	HLA-DMA, CD86, CD74
GO:0051251	5.0E-03	21.37	0.11	2	63	Positive regulation of lymphocyte activation	HLA-DMA, CD86
GO:0007626	5.5E-03	9.52	0.37	3	217	Locomotory behavior	CXCL9, CCL18, CXCL11
GO:0009605	5.6E-03	5.20	1.20	5	710	Response to external stimulus	IDO1, CXCL9, ORM1, CCL18, CXCL11
GO:0030097	5.6E-03	9.43	0.37	3	219	Hemopoiesis	HLA-DMA, CD86, CD74
GO:0002696	6.3E-03	18.88	0.12	2	71	Positive regulation of leukocyte activation	HLA-DMA, CD86
GO:0050867	6.6E-03	18.34	0.12	2	73	Positive regulation of cell activation	HLA-DMA, CD86
GO:0045321	6.8E-03	8.77	0.40	3	235	Leukocyte activation	HLA-DMA, CD86, CD74
GO:0048534	7.1E-03	8.66	0.40	3	238	Hemopoietic or lymphoid organ development	HLA-DMA, CD86, CD74
GO:0048583	7.2E-03	8.62	0.40	3	239	Regulation of response to stimulus	HLA-DMA, IDO1, CD86
GO:0050863	8.1E-03	16.47	0.14	2	81	Regulation of T cell activation	HLA-DMA, CD86
GO:0002520	8.4E-03	8.13	0.43	3	253	Immune system development	HLA-DMA, CD86, CD74

### Quantitative real-time PCR

Reverse transcription was performed using standard protocols. Quantitative Real-Time PCR (TaqMan) was carried out as described previously [18] using the following predesigned Assays-on-demand (Applied Biosystems) with beta-Actin as housekeeping gene: ACTB (Hs 030023880_g1), CD74 (Hs 00959496_m1), HLA-DMA (Hs 00157941_m1), CXCL11 (Hs 00171138_m1), CXCL9 (Hs 00970538_m1), IDO1 (Hs 00158032_m1). RT-PCR was run in a 7000 Sequence Detection System (Applied Biosystems) under the following conditions: 95°C for 10 minutes followed by 40 cycles of 95°C for 15 seconds and 60°C for 1 minute. Data analysis was performed according to the ΔΔC_t_ method wherein β-Actin was used as reference gene and colorectal carcinoma cells HT29 as calibrator [19]. Significance of the differences in gene expression between samples of patients with or without later metastasis as measured by qPCR were analyzed by Mann–Whitney test using SPSS 14 (SPSS, Chicago, IL, USA).

### Study design

This study includes retrospective cases, which were stratified as to contain mainly UICC II cases. Samples were taken from 1996 to 2004. Follow-up data were obtained for at least 41 months or until metastasis of disease with a follow-up time of 41–115 months (median: 77 months) for non-recurrent patients. The clinical endpoint examined was distant metastasis of disease. The number of samples initially available for this study was largely reduced because of the application of stringent quality criteria regarding sample characteristics, RNA and microarray quality (see above) and stringent criteria for the inclusion of patients.

## Results

### Microarray data analysis

Data analysis of gene expression profiles obtained by a comprehensive genome-wide gene expression study using oligonucleotide microarrays of 45 patients with CRC (Table 1) led to the identification of 44 probes that enabled us to discriminate between patients with distant metastasis during a follow-up time of 41 to 115 months after surgery and those who did not (Table 2). Of the 44 probes, 30 were associated with genes that had functional annotations including a gene symbol (using the hgug4112a annotation package for Bioconductor, version 2.2.11). GO-term enrichment analysis showed that the most significantly enriched terms were “immune system process”, associated with 14 genes, and “immune response”, associated with 13 genes, with the latter group being a subgroup of the former (Table 3). Only 11 of the top 47 most enriched GO terms were not directly related to immune system processes, and some of those 11 GO terms such as “response to stimulus”, “cell-cell signals”, and “taxis” might be also related to immune response mechanisms.

### Predictive value of the immune response signature

**Figure 1:**
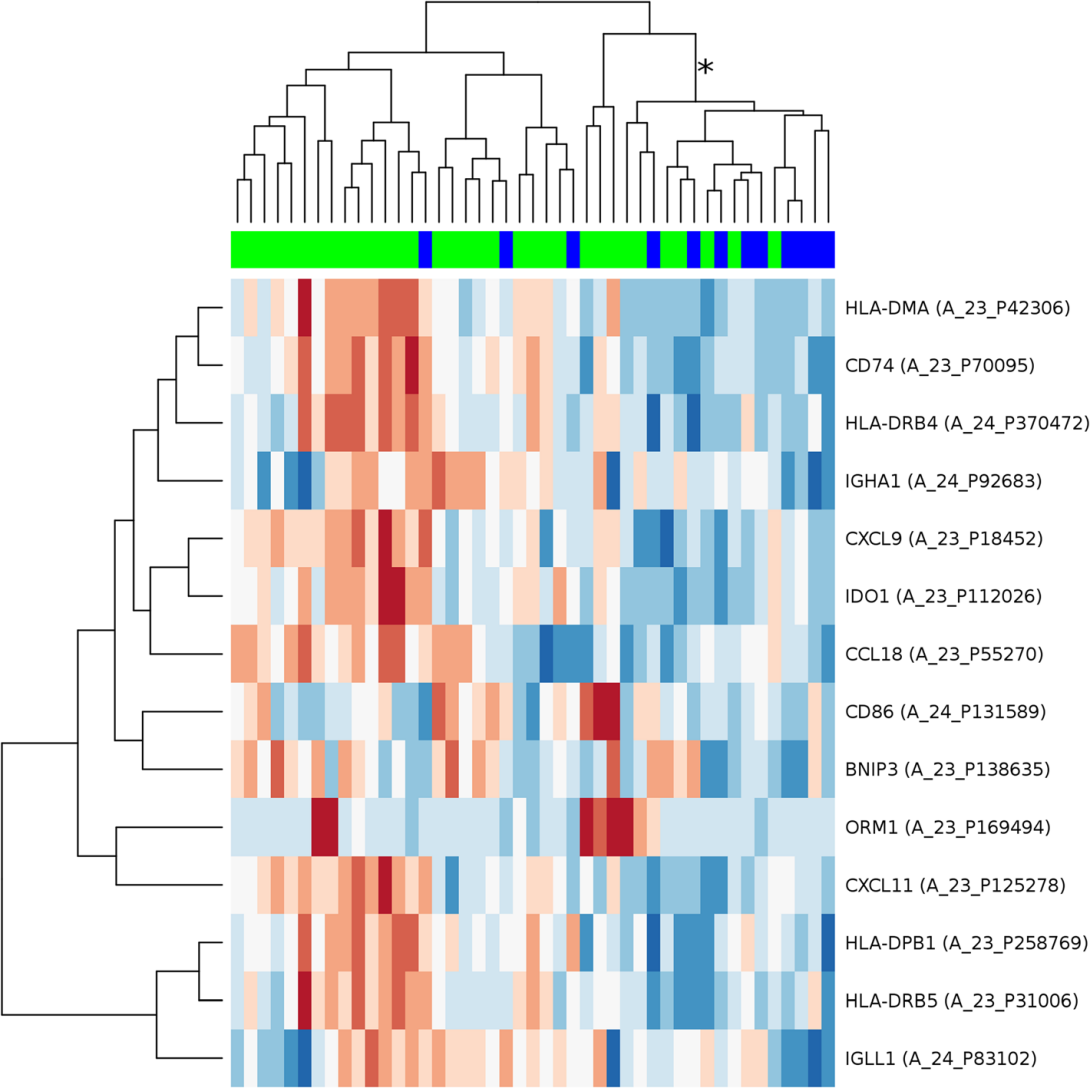
**Clustering of the cohort using 14 selected immune response genes.** Hierarchical clustering of 45 colorectal carcinomas, using a gene expression signature composed of 14 genes related to the immune system. Each row represents a probe set for a gene and each column a sample. Samples marked in blue are metastatic cases, samples marked in bright green are cases showing no metastasis at least 3 years after surgery. The length and the subdivision of branches display the relation of the samples based on their similarity in the expression of the 14 genes. Most of the metastatic cases are grouped in the branch marked with the asterisk (*). Metastatic cases have distinctly more of these 14 genes expressed at low expression values (blue, low expression) than non-recurrent patients (red, high expression). The probe hybridization values have been scaled and centered row-wise.

**Figure 2:**
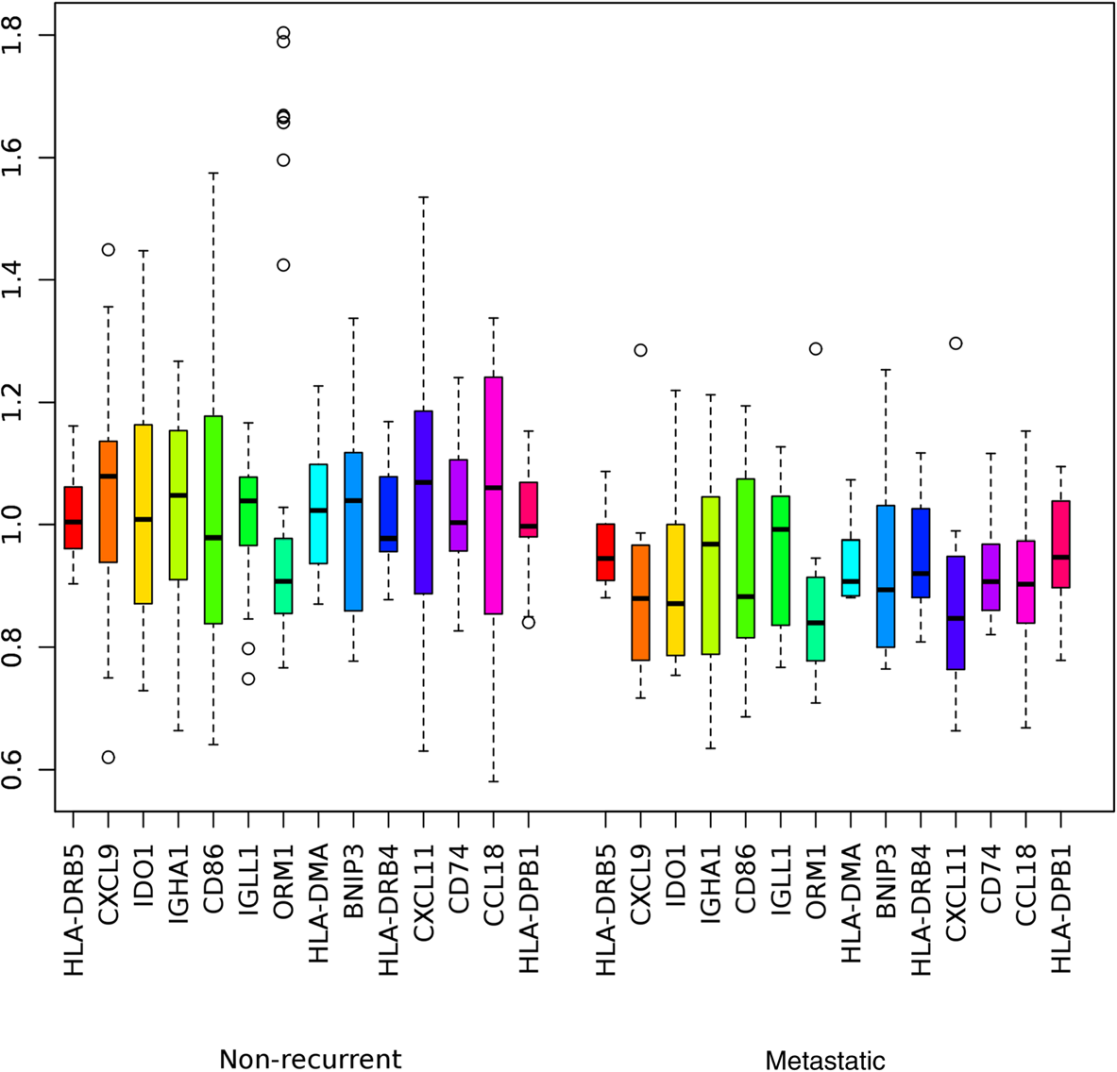
**Normalized expression values.** The expression values of the 14 immune system-related genes were normalized and median centered. Depicted are the normalized expression values for each of the immune response genes in non-recurrent (n = 33) and metastatic (n = 12) cases.

**Figure 3:**
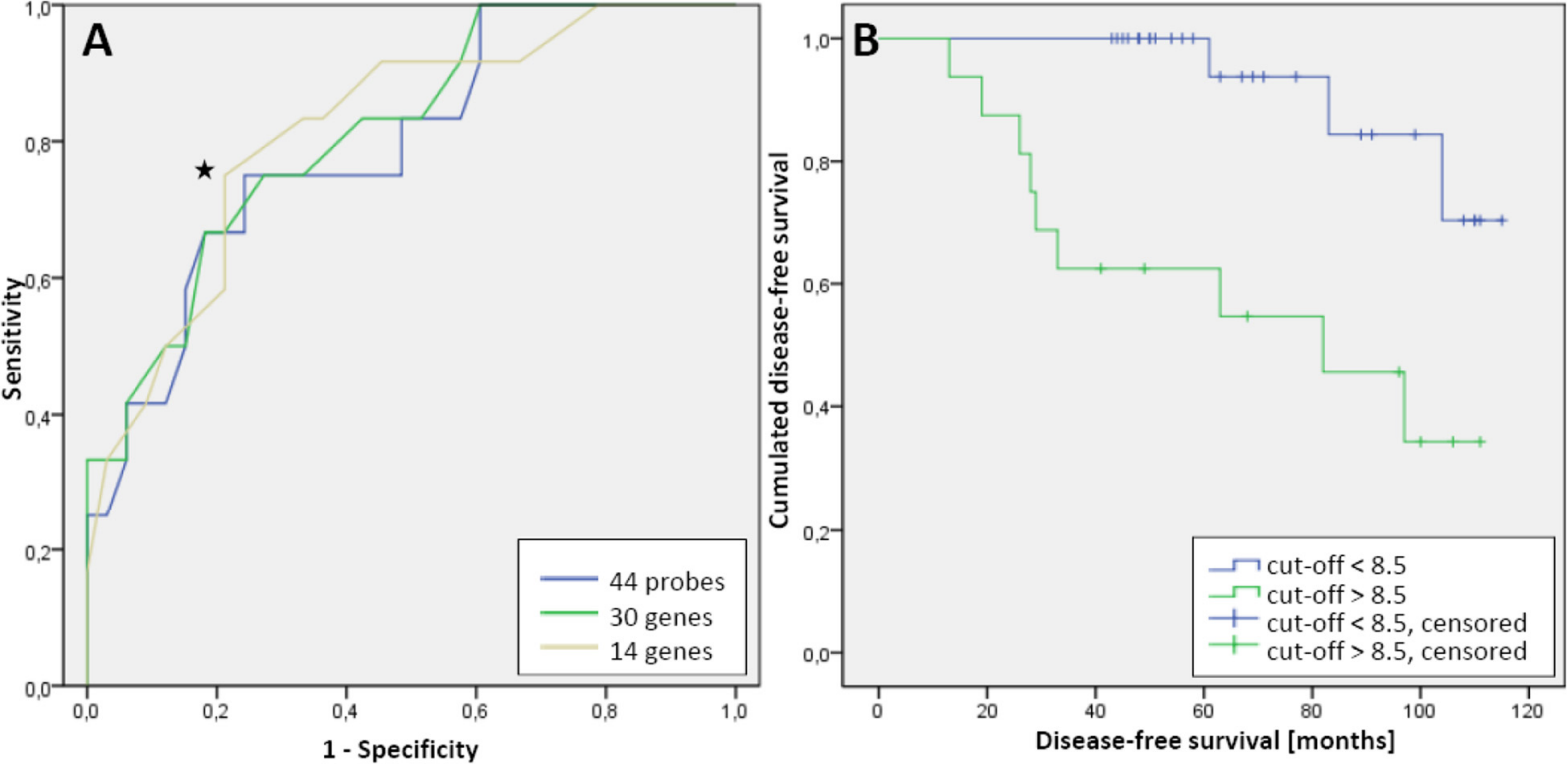
**Predictive value of the immune response signature. (A)** ROC-curve of a classifier for metastasis based on number of immune system related probes (brown curve, 14 genes) expressed below the median of the whole set. A cut-off of 8.5 probes (marked with ) was selected based on the ROC-curve shown in this figure (AUC = 0.817). This cut-off yields a specificity of 79% and a sensitivity of 75% (asymptotic significance = 0.001, asymptotic 95% confidence interval = 0.678 – 0.956). For comparison, ROC-curves using one probe for each of the 30 selected genes (related or not to immune response; green curve, 30 genes), or all their associated probes (one gene may have more than one associated probe; blue curve, 44 genes), were not very different. **(B)** Kaplan Maier-Curve showing cumulated disease-free survival vs. disease-free survival time using the cut-off of 8.5 as described in Figure 3; p = 0.002 (logRank Mantel-Cox), n = 45.

The 14 genes associated to immune system processes were used for further analysis. Hierarchical clustering of the hybridization values of the corresponding 14 probes produced a branch that consisted of a majority of patients with metachronous metastases (Figure 1). The analysis suggests that the metastasized cases have more of these 14 genes expressed at low levels than non-recurrent patients, although not always the same genes. In fact, all of the 14 genes in the immune related signature have average values of expression that are lower in the set of metastasized cases than in the set of non-recurrent patients (Figure 2). According to these observations, we devised a simple classifier using the hybridization values of the probes associated to the 14 immune system related genes. If at least a certain number of the 14 probes are below their median hybridization values in all other samples, then that sample is predicted to be metastatic. By ROC-curve analysis of this gene signature (Figure 3), a cut-off of 8.5 probes was selected. This means that if at least 9 of the putative marker probes show a value below their median expression value, then that sample is classified as a case that would suffer later from metachronous metastases. This cut-off yields a specificity of 79% and a sensitivity of 75%.

### Validation of the microarray results

**Figure 4:**
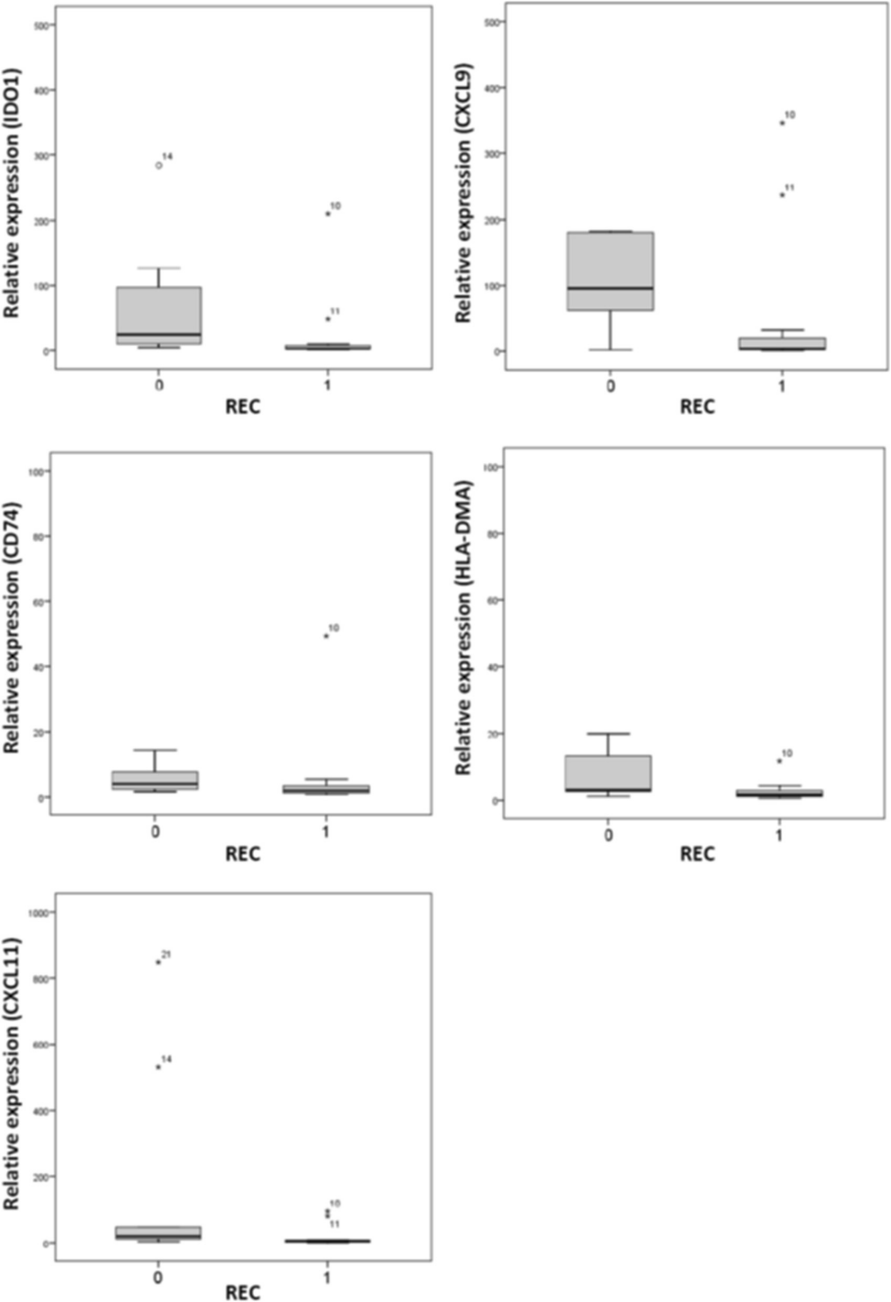
**Validation of the marker gene expression by quantitative RT-PCR.** Expression of five marker genes (IDO1, CXCL9, CD74, HLA-DMA, CXCL11) in primary colorectal carcinomas with (always on the right side, REC = 1, n = 11) or without later metastasis (left side, REC = 0, n = 11) as determined by qRT-PCR (TaqMan). Relative amount of expression is shown in Box – Whisker - Plots. Gray columns show a 50% range of the data surrounding the median; black lines within each column mark the median; asterisks mark outliers. Differences between REC = 0 and REC = 1 groups are significant (less than 5%), except for CD74.

For validation of microarray results qPCR was performed employing the five randomly chosen genes CD74, CXCL9, CXCL11, HLA-DMA and IDO1 from the 14 immune system related genes found by microarray analysis. Here, we used a carefully selected collection of the previously samples encompassing 11 patients which showed later metastasis and 11 patients without metastasis during the follow-up time which are similar in age, gender and tumor staging (Table 4). The results of the qPCR analysis showed that the expression of these genes is lower in cases with later metastasis for all of the examined genes, thereby confirming the microarray results (Figure 4). Differences in gene expression between both groups are significant (less than 5%), except for CD74 which shows only a trend into this direction.

**Table 4:** Patient characteristics of samples used for qPCR validation

**Characteristic**	**Non-recurrent**		**Metachronous metastasis**		**p-value**^**1**^
	**absolute**	***%***	**absolute**	***%***	
**Total number**	11		11		
**Age [years]**					n.s.
Mean	68		67		
**Gender**					n.s.
Female	7	*63,6*	7	*63,6*	
Male	4	*36,3*	4	*36,3*	
**UICC stage**					n.s.
I	2	*18,2*	2	*18,2*	
II	8	*72,7*	7	*63,6*	
III	1	*9,1*	2	*18,2*	

## Discussion

One characteristic feature of the current study is its stringency with regard to sample collection and RNA/microarray quality controls. Primary CRC can be divided into two main groups, firstly tumors with chromosome instability (CIN) showing high rates of chromosome losses and gains and secondly tumors with microsatellite instability (MSI) characterized by genome-wide changes in repetitive sequences due to defects in the DNA mismatch repair system [20]. Since these two groups of CRC also differ in many aspects including survival of the patients [21], microsatellite instability of all tumors was tested and MSI samples were excluded. This strategy thus strengthens sample homogeneity.

Using a novel way of microarray data analysis (see Data analysis section) a gene signature containing 44 probe sets was identified (Table 2), which was predictive of cancer metastasis. Almost half of the genes were related to immune system processes and immune response. Obviously, one may assume that not only the specific characteristics of the tumor cells themselves, such as angiogenesis, invasion, and proliferation or apoptosis, are relevant for metastasis but also the immune response of the host organism. In fact, it is clear that the host immune response is an important factor affecting cancer progression [22]. However, the complex interplay between the tumor’s immunogenicity, the host’s immune response and the cancer cells’ capabilities of evading immune response is still a challenging subject of current research. CRCs are immunogenic and provoke the host’s immune response, its strength being related to the patient’s prognosis [23]. High concentrations of neutrophils [24], high preoperative levels of natural killer cells [25], high numbers of mast cells [26,27], a high percentage of CD4^+^ T cells [28], the infiltration of the tumor with high numbers of central memory T cells and high CD8^+^ T cell counts [29], for example, have been found to correlate with a better prognosis. Yet, under the selective pressure of the immune system, cancers “escape” by becoming less immunogenic e.g. by failing to express MHC class I antigens – a process called immunoediting [30]. Apart from that, there is evidence that the cancer itself has a direct immunosuppressive effect, which starts at the molecular and cellular level and may lead to a basal shift in immune function [31].

Here, our analysis showed that our prognostic gene signature of 44 genes includes 14 genes related to immune response (Figure 1, Figure 2) that are down-regulated in primary carcinomas which later metastasize. In general, we hypothesize that the reduced expression of these 14 prognostic immune response genes impedes activation of CD4 T-cells, mostly involving the MHC class II pathway. Each of the 14 genes’ roles in this process is discussed in the following section. Validation experiments using qPCR confirmed downregulation of CD74, CXCL9, CXCL11, HLA-DMA and IDO1.

### Reduced expression of class II molecules might impede activation of CD4 T cells

The ability of the immune system to recognize and defend against the introduction of foreign antigens depends on the ability of the host’s MHC class II, which presents peptides degraded in intracellular vesicles to circulating CD4 T cells. Class II molecules such as HLA-DMA are immunological proteins vital to the proper loading and presentation of these peptides in macrophages, immature dendritic cells, B cells, and other antigen presenting cells. HLA-DPB, HLA-DRB4, and HLA-DRB5 also belong to this group. Here, a downregulation of components of the MH) class II was found in primary carcinomas of patients with later metastasizing tumors. In accordance, Lovig et al. [32] found that patients positive for the HLA-DR determinants showed better survival than those without HLA-DR expression. Similarly, in MSI tumors with a good prognosis an upregulation of HLA-DMA was noted [33]. Furthermore, HLA-DPB1 has been found to be downregulated in metastasizing primary tumors of different origins, including colorectum [34]. Down-regulation of four MHC class II molecules has been reported to be significant for primary tumors of hepatocellular carcinoma which show later metastases [35]. Recently, the presence of inactivating mutations in the HLA-A gene was reported for squamous lung carcinomas [36].

### Reduced expression of CD74, CD86, and CCL18 might impede activation of CD4 T cells

CD74 (HLA-DR-associated invariant chain) plays a critical role in MHC class II antigen processing by stabilizing peptide-free class II alpha/beta heterodimers in a complex soon after their synthesis and directing transport of the complex from the endoplasmic reticulum to compartments where peptide loading of class II takes place. CD86 (Cluster of Differentiation 86) is expressed on antigen-presenting cells that provide co-stimulatory signals necessary for T cell activation and survival [37]. CCL18 is relevant for activation of T-cells through MHC class II, and is a marker for tumor-associated macrophages [38]. Moreover, it is a prognostic marker in gastric cancer and probably plays a role in the physiological homing of lymphocytes and dendritic cells as well as in the generation of primary immune responses [39,40].

### Reduced expression of CXCL9 and CXCL11 might impede activation of T cells

CXCL9 and CXCL11 (Chemokine (C-X-C motif) ligands 9 and 11) are small cytokines belonging to the CXC chemokine family also known as “Monokine induced by gamma interferon” [41]. They are produced by three different cell types, monocytes, endothelial cells and fibroblasts, play a role as T-cell chemoattractants and are secreted in response to IFN-γ. Chemokines CXCL9, CXCL10 and CXCL11 are closely related. All three genes are located on human chromosome 4 and they all elicit their chemotactic functions by interacting with the chemokine receptor CXCR3.

### A conflicting result concerning IDO1 (Indoleamine-2,3-Dioxygenase)

Elevated tryptophan catabolism in the urine and blood of tumor-bearing patients has been recognized for many decades. For example, biopsy samples taken from CRC patients show an overexpression of IDO1 [42]. Although expression did not correlate with patient survival, increased IDO1 expression did correlate with liver metastasis. However, another study in patients with HCC showed that IDO1 expression in tumor specimens was positively correlated with progression-free survival [43]. Moreover, a significant inverse correlation between the density of IDO1-positive microvessels and the number of proliferating tumor cells in primary and metastatic renal cell carcinoma was found [44]. Increased expression of IDO1 in endothelial cells of tumors correlated positively with long-term patient survival. Thus, these data suggest that IDO1 can limit tumor growth. Similarly, in our study we found an increase of IDO1 expression in primary carcinomas with good prognosis. The opposing effects of IDO1 have been discussed recently [45].

Enrichment in immune response functions among the set of genes of prognostic relevance is in agreement with other studies. CXCL9 and CXCL11 – as well as IDO1 - are part of a published prognostic signature that predicts metastasis in CRC [46]. Using Affymetrix arrays Lin *et al.* aimed to develop gene classifiers to predict colorectal cancer metastasis. Eleven of 19 genes in the classifier were involved in the immune response. In agreement with our results all of the 11 immune response genes were down-regulated in metastatic cases [46]. Accordingly, a comprehensive study using different assays with the aim to elucidate the mechanisms underlying immune response in CRC showed that a high expression of CXCL9 and CXCL10 is correlated with a favorable outcome of this disease [47]. Furthermore, CXCL9 and IDO1 have been shown to be prognostic markers in breast cancer [48]. Only recently, 15 immune response genes, among them IGHA1, IGHG1 and IGL@ were found to be part of a 128 genes signature that predicted metastasis in CRC [8].

## Conclusions

Whereas up to now only gene signatures containing genes of various biological functions have been described for prediction of metastasis in CRC, in this study metastasis could be well predicted by a set of gene expression markers consisting exclusively of genes related to the MHC class II complex clearly involved in immune response. From our data we cannot state whether the later recurring tumor is the cause or the beneficiary of the suppressed immune response. Nevertheless, our data show that the proper function of a comprehensive network of immune response genes is of vital importance for the survival of colorectal cancer patients. Recent results indicating that the tumor microenvironment can reduce the maturation of dendritic cells [49,50] hint to the importance of our findings and suggest avenues for prognosis and treatment.

## Abbreviations

CRC: Colorectal cancer; GO: Gene ontology; SVM: Support vector machine.

## Competing interests

The authors declare that they have no competing interests.

## Authors’ contributions

Conception and design: MAA and WK; data acquisition: MF and TJ; data analysis and interpretation: MRH, MF, TJ and WK; manuscript writing: MF,MAA and WK. All authors read and approved the final manuscript.

## Pre-publication history

The pre-publication history for this paper can be accessed here:

http://www.biomedcentral.com/1471-2407/14/64/prepub

## Supplementary Material

Additional file 1: Figure S1 Principal component analysis of the microarray data used in this study. Click here for file
