# Supplementary figures and images for: Concerted down-regulation of immune-system related genes predicts metastasis in colorectal carcinoma
Source: BMC Cancer. 2014 Feb 5;14:64. doi: 10.1186/1471-2407-14-64 (PMC3922093; doi:10.1186/1471-2407-14-64)

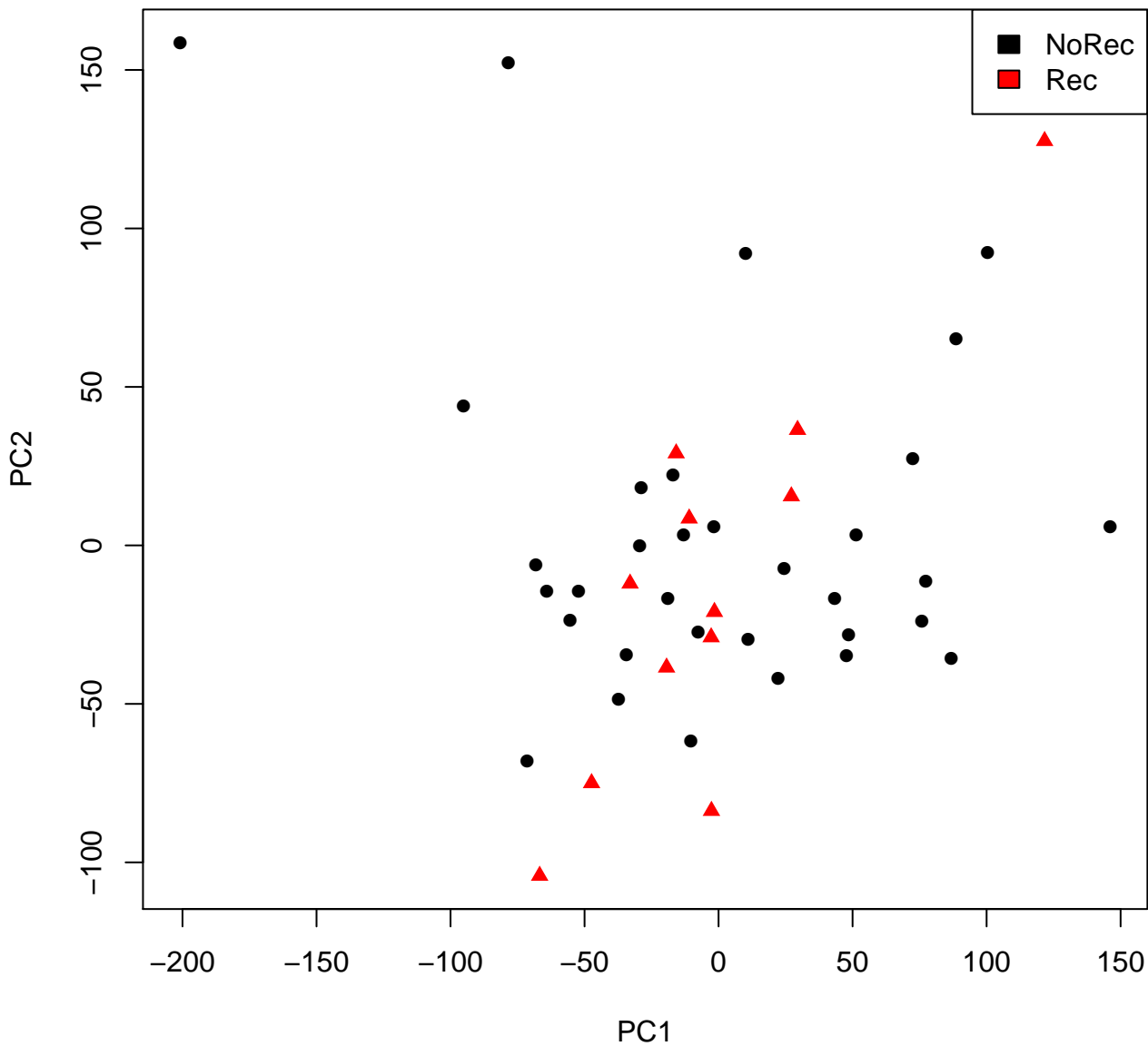

Supplement: Additional file 1: Figure S1 — Principal component analysis of the microarray data used in this study. [file 1471-2407-14-64-S1.pdf]
